# Passively mode-locked interband cascade optical frequency combs

**DOI:** 10.1038/s41598-018-21504-9

**Published:** 2018-02-20

**Authors:** Mahmood Bagheri, Clifford Frez, Lukasz A. Sterczewski, Ivan Gruidin, Mathieu Fradet, Igor Vurgaftman, Chadwick L. Canedy, William W. Bewley, Charles D. Merritt, Chul Soo Kim, Mijin Kim, Jerry R. Meyer

**Affiliations:** 1 Jet Propulsion Laboratory, California Institute of Technology, Pasadena, CA 91109 USA; 2 Naval Research Laboratory, Washington, DC 20375 USA; 3 Sotera Defense Solutions, Inc., Columbia, MD 21046 USA

## Abstract

Since their inception, optical frequency combs have transformed a broad range of technical and scientific disciplines, spanning time keeping to navigation. Recently, dual comb spectroscopy has emerged as an attractive alternative to traditional Fourier transform spectroscopy, since it offers higher measurement sensitivity in a fraction of the time. Midwave infrared (mid-IR) frequency combs are especially promising as an effective means for probing the strong fundamental absorption lines of numerous chemical and biological agents. Mid-IR combs have been realized via frequency down-conversion of a near-IR comb, by optical pumping of a micro-resonator, and beyond 7 *μ*m by four-wave mixing in a quantum cascade laser. In this work, we demonstrate an electrically-driven frequency comb source that spans more than 1 THz of bandwidth centered near 3.6 *μ*m. This is achieved by passively mode-locking an interband cascade laser (ICL) with gain and saturable absorber sections monolithically integrated on the same chip. The new source will significantly enhance the capabilities of mid-IR multi-heterodyne frequency comb spectroscopy systems.

## Introduction

Optical frequency combs in the visible to near-infrared range of the electromagnetic spectrum have quickly become standards for precise measurements of frequency and time^1–4^, besides having revolutionized precision spectroscopy^5–9^. Frequency combs are also finding application in attosecond science, optical waveform generation, remote sensing, microwave synthesis, optical communications and astrophysics. The extension of frequency combs into the midwave infrared (mid-IR) has broad implications for molecular composition spectroscopy, since numerous molecules undergo strong vibrational transitions in this range. Optical combs can provide precision spectroscopy^10,11^, with large dynamic range in real-time. In particular, mid-IR combs can be exploited to detect small traces of environmental and toxic agents in atmospheric, security, and industrial applications, because a mid-IR beam can propagate over long distances in the earth’s atmosphere with small attenuation. To date, most mid-IR combs have been realized via frequency down-conversion of a near-IR comb through optical parametric oscillation^12,13^ or difference frequency generation^14–17^ or by continuous wave (cw) optical pumping of a micro-resonator^18–20^. Beyond 7 *μ*m, frequency combs based on four-wave mixing in quantum cascade lasers (QCLs) have recently produced high output powers with wide optical bandwidth^21,22^. However, QCL performance degrades in the 3–4 *μ*m band where a large fraction of the absorption features associated with C-H bonds are clustered. By mode locking a new class of semiconductor laser, we have demonstrated the first electrically pumped optical frequency combs to operate in the 3–4 *μ*m wavelength range. The combs generate sub-picosecond pulses at gigahertz repetition rates.

## Mode-locked interband cascade lasers

**Figure 1:**
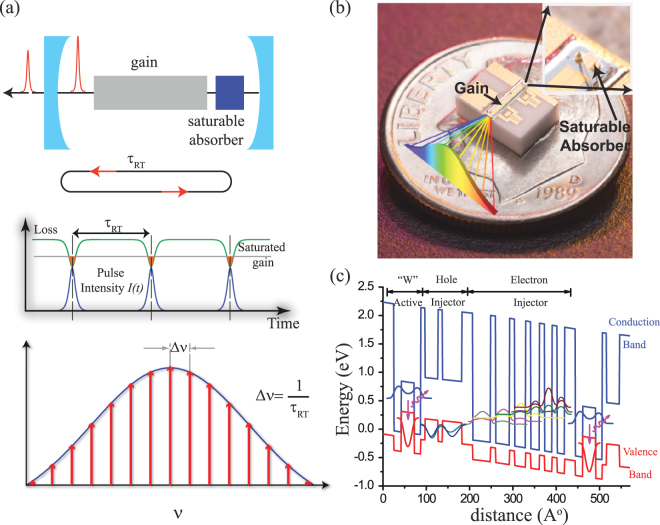
Passively mode-locked laser. (**a**) Schematic of a laser cavity with gain and saturable absorber sections (top panel). The saturable absorber imposes pulsed operation by providing an intensity-dependent loss mechanism (middle panel). The generated pulses form an optical frequency comb with modes separated by the cavity’s free spectral range, FSR (bottom panel). (**b**) An ICL mode-locked laser consisting of gain (front) and saturable absorber (back) sections. (**c**) A typical ICL band diagram, 33 with the conduction band in blue, valence band in red, and wavefunctions for several key electron and hole states.

Mode-locked lasers have frequently been used to generate frequency combs in the visible and near-IR. A phase-coherent train of very short pulses (<1 ps) generated by a mode-locked laser at a rate equal to the round-trip time in the optical cavity (*τ*_*RT*_ = L/*ν*_*g*_, where *ν*_*g*_ is the group velocity and L is the round-trip cavity length) translates into a comb spectrum consisting of discrete, regularly spaced optical modes, as shown in Fig. 1(a). Output in a very short pulse is generated by maintaining a well-defined phase relationship among multiple axial modes in the laser cavity. In a monolithic passive mode-locking architecture, this relationship is imposed by an intra-cavity, intensity-dependent loss element, the saturable absorber (SA). Such lasers can be formed monolithically in a split-contact gain/saturable-absorber architecture, in which the top contact is divided into a longer, forward-biased gain section and a shorter SA section that may or may not be biased (usually in reverse), Fig. 1(b)^23^. In the visible and near IR, several variations on this geometry have generated ultra-short (sub-picosecond) transform-limited pulses with high repetition rates (terahertz) and low jitter, for optical communication and other applications.

Quantum cascade lasers (QCLs) have produced frequency combs at longer wavelengths extending from 7 *μ*m into the THz range^21,22,24^. However, the extremely short (<1 ps) upper-state lifetime of the QCL’s intersubband-based gain process makes passive mode-locking intrinsically difficult. Hence all of the QCL frequency combs reported to date have relied on four-wave mixing due to a large third-order nonlinearity (*χ*^3^) to generate frequency-modulated (FM) output with little or no intensity modulation. Besides, QCLs tend to perform less favorably at wavelengths shorter than ≈4 *μ*m because of heavy strain and limited conduction band offsets in the strain-compensated InGaAs/InAlAs structures on InP^25^.

In the present work we demonstrate an electrically pumped frequency comb centered at $\lambda \sim 3.6$ *μ*m, which is produced by passively mode locking a mid-IR interband cascade laser (ICL). The ICL may be considered a hybrid of a conventional diode laser, in that the optical gain is due to interband transitions, and a QCL, in that a staircase of active stages are stacked in series to reduce the threshold current density and parasitic voltage drop^26,27^. While at *λ* > 4 *μ*m the maximum output power tends to be higher for a QCL^28,29^, ICLs spanning 3–6 *μ*m are ideal for many low-power spectroscopic applications since cw operation at room temperature consumes an order of magnitude less drive power than a QCL emitting at the same wavelength^30,31^. This is especially advantageous in battery-powered sensing systems, or for environments requiring small size and weight.

Figure 1(b) illustrates the 4-mm-long laser cavity with split contacts that was used in this study. The ICL wafer with 7 active stages was grown by molecular beam epitaxy on an n-GaSb substrate, using design and growth procedures similar to those discussed previously^32^. The laser cavity, processed as described in Methods below, features a 200-*μ*m-long saturable absorber section that is separated from the gain section by a non-contacted 100-*μ*m-long gap to prevent electrical shorting of the two sections. For this architecture to be effective, the material gain recovery time (*τ*_*g*_) should exceed the cavity round trip time, *τ*_*RT*_, which is satisfied in an ICL due to the long carrier lifetime for interband transitions (*τ*_*g*_ ≈ 500 ps, vs. *τ*_*RT*_ ≈ 100 ps for a 4 mm long cavity)^23^. Furthermore, the time required for the SA section to recover strong absorption (*τ*_*abs*_) should be shorter than the gain recovery time. These conditions open an amplification window around the pulse because rapid absorption recovery shortens the leading edge of the pulse. While the same process can also shorten the trailing edge if *τ*_*abs*_ is shorter than the pulse width, the present devices do not fall in the fast SA regime.

Several schemes are commonly used to accelerate the absorption recovery in a mode-locked diode laser that monolithically incorporates a saturable absorber. These include ion implantation and the use of a split contact to reverse bias the SA section. In our demonstration, *τ*_*abs*_ was reduced by implanting high-energy H^+^ ions (see Suppl. Mat. Section 1). A split contact was also patterned to allow separate biasing of the SA section, although that feature was not used in the experiments reported here and the SA junction was left open.

## Mid-IR optical frequency combs

**Figure 2:**
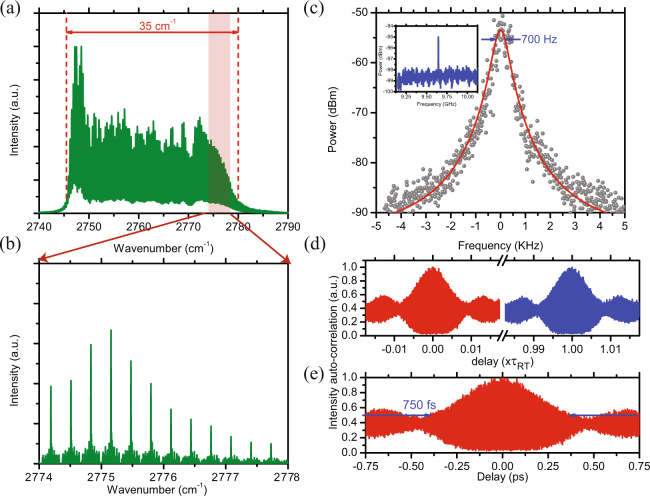
Passively mode-locked interband cascade laser (ICL) (**a**) FTIR spectrum of the mode-locked ICL operating at I = 325 mA and T = 15 °C with the SA junction left open, showing >35 cm^−1^ (1.05 THz) bandwidth and >120 modes. (**b**) A breakout of the mode-locked spectrum measured by a high-resolution FTIR (100 MHz) under the same operating conditions as (**a**). (**c**) RF beat-note with 700 Hz linewidth. The inset shows the RF tone at 9.68 GHz on a much broader scale. (**d**) Intensity autocorrelation trace collected using two-photon absorption in an extended InGaAs detector (See Supp. Mat. 3). The presence of a dominant peak at delay times corresponding to multiples of the cavity round trip time, superimposed with the zero delay peak, confirms that the short pulses circulating in the optical cavity are mode-locked. (**e**) The trace shows that the mode-locked ICL emits pulses of width ≈750 fs.

Under dc bias, the laser operates at T = 15 °C with a threshold of I _*th*_ = 85 mA (see Suppl. Mat. Section 2). Although just above threshold the emission is into a single longitudinal mode, at higher currents the spectrum broadens (>1 THz) and becomes multi-mode. Figure 2(a) shows the lasing spectrum at 325 mA and 15 °C, as acquired by a Fourier transform infrared (FTIR) spectrometer (0.075 cm^−1^, 2200 MHz). The overall bandwidth is 35 cm^−1^ (52 nm), while Fig. 2(b) breaks out a smaller frequency range that was measured with a high-resolution FTIR (0.0033 cm^−1^, 100 MHz). The comb emission comprises over 120 modes, with >50 *μ*W of power in each mode.

In this geometry the saturable absorber section functions as a fast optical detector that produces an electrical RF tone useful to analyze the laser performance and synchronize external events. The same Fabry-Perot cavity with no coupling among its axial modes will display an RF tone near the round-trip frequency (Δ*ν* in Fig. 1(b)) with >1 MHz linewidth due to relative phase fluctuations of the incoherent modes. However, Fig. 2(c) shows a far narrower RF spectrum with linewidth only 700 Hz for the multi-section ICL operating at I = 325 mA. The narrow linewidth, large signal-to-noise ratio, and stability of this beat note confirm that mode locking has been achieved, with negligible random drift of the relative phases of the >120 modes lasing simultaneously in the cavity.

Under mode-locked conditions, the axial modes in the Fabry-Perot cavity combine coherently to generate very short optical pulses. We have employed second-order auto-correlation to analyze the temporal characteristics of the pulses. In this measurement, the optical pulse is combined with its delayed replica, and the combined beam is sent collinearly into an extended InGaAs photodiode in which it is detected by two-photon absorption. While the laser photon energy corresponding to *λ* = 3.6 *μ*m (0.34 eV) is insufficient to bridge the detector bandgap of 0.59 eV, two-photon absorption in the InGaAs generates a second-order autocorrelation spectrum (Suppl. Mat. Section 3). Figure 2(d) shows the measured second-order autocorrelation for the mode-locked ICL operated at I = 325 mA and T = 15 °C. The autocorrelation spectrum shows a strong peak at zero delay, which is expected since with no delay the two beams should overlap perfectly. The presence of a dominant peak at delay times corresponding to multiples of the cavity round trip time, superimposed with the zero delay peak can only occur when the pulses from the two arms overlap temporarily on the detector and confirms that the short pulses circulating in the optical cavity must be mode-locked^33^. The full-width at half maximum (FWHM) of the narrow peak at zero delay is less than 1 ps. That the interferogram signal does not disappear entirely outside the main peak implies the presence of parasitic modes that are not phase-locked to the rest of the modes traveling in the cavity and as a result the peak-to-background ratio deviates from the expected 8:1 ratio for a mode-locked laser^34^.

## Multi-heterodyne beating of ICL combs

**Figure 3:**
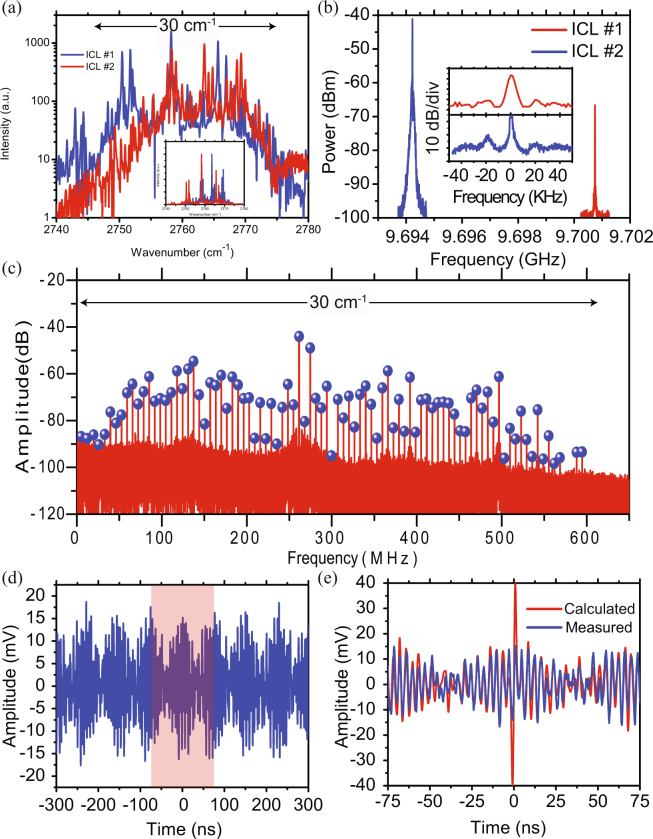
Heterodyne beat note experiment (**a**) FTIR spectra of two closely matched (Δf _*rep*_ = 6.5 MHz) mode-locked ICLs operating at I = 304 mA and T = 15.4 °C (ICL #1), and I = 287 mA and T = 13.3 °C (ICL #2) spanning 30 cm^−1^. (**b**) Intermode beat notes (RF tones) measured electrically from the saturable absorber section. The inset shows a zoom-in view of the measured spectra with 2 KHz and 4 KHz linewidths for the blue and red spectra, respectively. (**c**) Phase-corrected (coherently averaged) multi-heterodyne spectrum extracted from a beating signal acquired over 100 *μ*s. (**d**) Unprocessed time domain interferogram showing 4 periods of the optical beating. (**e**) Zoom into the central part of (**d**), showing a comparison of one period of the measured beating signal with a simulation assuming that all modes in (**c**) have equal phases (ΔΦ = 0). Its shape is determined mainly by the two strongest beat notes in the RF spectrum at 260–270 MHz in (**c**).

To further explore the comb operation, we performed a multi-heterodyne beat note experiment in which the outputs of two distinct free-running mode-locked ICLs, spanning more than 30 cm^−1^ of optical bandwidth, were combined and detected by a fast mid-IR detector (HgCdTe, 0.8 GHz 3-dB bandwidth, VIGO). Figure 3(a) and (b) show the individual ICL optical and electrical beat-note spectra detected for the two lasers. Note that these devices were chosen for their suitable mode overlap, even though their comb amplitudes are less uniform than in the spectrum of Fig. 2(a) and the beat-note linewidths are slightly broader than in Fig. 2(c). The small difference of 6.53 MHz (0.067% of the center frequency) between the two repetition rates is achieved via precise microfabrication control and tuning of the operating conditions of the two lasers. This difference in the repetition frequency allows us to map 0.90 THz of optical bandwidth into 600 MHz of electrical spectrum, as shown in Fig. 3(c). The RF tone linewidths measured in a multiheterodyne experiment are smeared by the line-to-line frequency fluctuations of two independently free-running optical frequency combs. Such offset fluctuations in the optical mode frequencies which are common in frequency combs (e.g. QCL combs^35,36^ and fiber laser combs^37^), translate into multiplicative common phase noise. Hence, the corresponding time-domain interferogram, acquired within 100 *μ*s, was pre-processed prior to calculating the Fourier Transform by using a phase-correction technique recently demonstrated for spectroscopy with noisy Fabry-Perot ICLs to improve the beat note signal-to-noise ratio (SNR)^38^. The procedure increases the beat note signal-to-noise-ratio (SNR) by approximately 15 dB, and apparently also reduces their linewidth by compensating for slight fluctuations of the carrier-envelope offset and repetition frequencies of the combs, which distribute the amplitude of the down-converted electrical signal over a range of frequencies (see Suppl. Mat. Sections 4 and 5).

Figure 3(d) shows four periods of the time-domain beating signal with significant amplitude modulation. It is to be noted that the repetition rate of 6.5 MHz for the optical bursts is within the cut-off frequency for thermal effects in semiconductor laser diodes (1–10 MHz)^39^. Therefore thermal fluctuations in the two independently-running ICL combs could result in the shot-to-shot fluctuations observed in the measured interferogram trace. While Fig. 3(e) shows a simulation wherein the beat notes (Fig. 3(c)) were assumed to have constant amplitudes and equal phases (ΔΦ = 0)^40^. The simulation shows good agreement with measured interferogram (see Suppl. Mat. Section 6 for details).

In summary, we have demonstrated the first passively mode-locked mid-IR semiconductor lasers. The electrically-pumped interband cascade devices with gain and saturable absorber sections monolithically patterned onto the same ridge emit near 3.6 *μ*m, with a frequency comb bandwidth of 35 cm^−1^. For operation with a dc bias at 15 °C, the pulse length is <1 ps and the RF beat-note linewidth is <1 kHz. The beating of two combs with slightly different repetition frequencies is also demonstrated. This new capability offers unique opportunities for broadband laser spectroscopy to probe the strong fingerprint absorption lines of numerous chemical and biological agents. A focus of future work will be to increase the spectral width of the comb output, by using chirped quantum-well thicknesses in the ICL active stages to broaden the gain spectrum. The reported devices exhibit mode-locking only over relatively narrow ranges of current and temperature due mainly to the significant group velocity dispersion present in this generation of ICL waveguides. Our team is currently working to increase the locking range and operating conditions by managing the dispersion in the ICL waveguides.

## Device Fabrication

The first step is to bombard the saturable absorber sections of the ICL ridges with 350 keV H^+^ ions at a dose of 5 × 10^13^ cm^−2^. A 3-*μ*m-thick gold mask prevents penetration of the high-energy ions into the gain sections. Subsequently, narrow-ridge waveguides are defined by contact lithography and transferred onto the wafer surface by plasma etching. The ridge widths of <4 *μ*m support only a single lateral mode. To prevent current spreading, the etch that defines the ridges proceeds to a depth below the active stages. The plasma etch recipe produces ridge waveguides (RWGs) with reduced roughness and near-vertical sidewalls. Current is injected via a narrow metal contact patterned on the top surface of each ridge. For insulation, a thin dielectric film covers the ridge sidewalls, after which 3 *μ*m of gold is electroplated on top to provide heat extraction and allow wire bonding.

The epi-side processing yields 4-mm-long narrow RWGs with metal contact stripes on top. The gain section comprises more than 90 % of the total cavity length, with the saturable absorber and non-contacted divider of width ≈100 *μ*m occupying the rest. The gap between the gain and SA top contacts allows each to be biased individually.

The wafer is next polished to a thickness of ≈100 *μ*m to facilitate cleaving into individual lasers with mirror-like end facets. After backside polishing, a conformal metal contact surface is deposited on the back and then annealed for ohmic contact.

## Measurement

Neither facet of the cleaved single-element devices is coated. The lasers are mounted epitaxial-side up on gold-plated BeO submounts to improve the heat extraction and facilitate measurement. The laser output is collimated using a high-numerical-aperture lens (NA = 0.75), and then measured with a calibrated thermopile detector.

The lasing spectra are measured using a Fourier transform infrared (FTIR) spectrometer with 0.0033 cm^−1^ (100 MHz) resolution. The RF beat note spectrum is collected through a bias-T that is connected to the saturable absorber contact section.

## Electronic supplementary material

Supplementary Information
