# Supplementary material for: Passively mode-locked interband cascade optical frequency combs
Source: Sci Rep. 2018 Feb 20;8:3322. doi: 10.1038/s41598-018-21504-9 (PMC5820280; doi:10.1038/s41598-018-21504-9)
Supplement: Supplementary file 1 — Supplementary Information [file 41598_2018_21504_MOESM1_ESM.pdf]

Passively mode-locked interband cascade optical frequency combs

Mahmood Bagheri,[1] Clifford Frez,[1] Lukasz A. Sterczewski[1], Ivan Gruidin,[1] Mathieu Fradet,[1] Igor Vurgaftman,[2] Chadwick L. Canedy,[2] William W. Bewley,[2] Charles D. Merritt,[2] Chul Soo Kim,[2] Mijin Kim[3] and Jerry R. Meyer[2]

[1]*Jet Propulsion Laboratory, California Institute of Technology, Pasadena, CA 91109, USA*

[2]*Naval Research Laboratory, Washington, DC 20375, USA*

[3]*Sotera Defense Solutions, Inc., Columbia MD 21046, USA*

Supplementary Information

1. H^+ implantation of ICL devices

When light traveling in the SA section of a split-contact RWG laser is absorbed, the resulting photo-carriers temporarily bleach the transmission to produce pulsed operation. However, following passage of the optical pulse the saturable absorber must quickly recover to the strong-absorption state if the passive mode-locking is to be stable.[1] Proton bombardment is commonly used in semiconductor-based saturable absorbers to satisfy this condition[2], since impurities and defects increase the non-radiative recombination rate. The recovery time can also be reduced by applying a reverse bias, which rapidly sweeps carriers out of the SA[3].

The carrier lifetime (τ_{car}) in an ordinary ICL is determined by Auger recombination and is much too long (~ 1 ns) to provide effective recovery. Therefore, the saturable absorber sections of the present devices were implanted with H^+ ions to significantly increase the recombination rate due to Shockley-Read-Hall recombination at the induced defects in the crystal structure. To study the effect of bombardment on the lifetime, photoluminescence experiments were performed on a series of ICL test structures that were implanted with various doses of 50 keV H^+ ions. At low excitation levels, it is reasonable to assume that the non-radiative carrier lifetime is roughly proportional to the PL intensity[4]:

$$I_{PL} \propto G\tau_{car}$$

where G is the carrier generation rate that depends on pump intensity. The comparison of the PL spectra for non-bombarded (red) and bombarded (10^{10} cm^{-2}, blue) structures in Fig. S1(a) shows that even a relatively low dose is sufficient to decrease the intensity. Figure S1(b) plots the PL intensities (relative to the non-bombarded value) at both 78 K (blue) and 300 K (red) for the entire series of samples with bombardment doses varying by three

orders of magnitude. As expected, the scaling with dose is approximately linear. The data imply that at 300 K, exposure of the IC active stages to a 10^{12} cm^{-2} dose reduces the carrier lifetime by > 100.

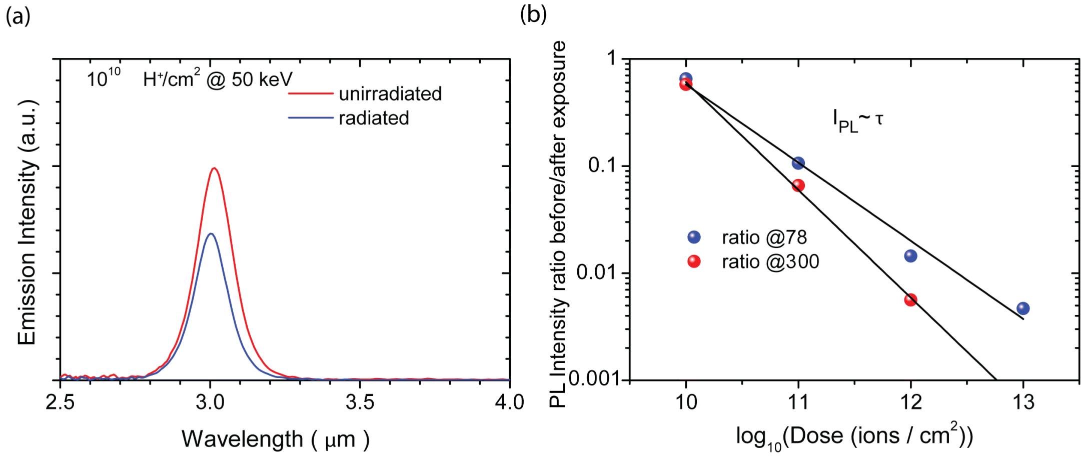

Fig. S1 (a). Room temperature photoluminescence spectra for non-bombarded (red) and bombarded (10^{10} cm^{-2} H^+) interband cascade test structures, plotted on the same uncalibrated intensity scale. (b) Bombarded to non-bombarded PL intensity ratio as a function of H^+ dose at 78 K (blue) and 300 K (red).

To more directly quantify the relation between bombardment dose and carrier lifetime, we next bombarded a series of broad-area interband cascade lasers, which were previously processed from the same chip, with doses of 50 keV H^+ ions ranging from $2x10^{12}$ to $6x10^{13}$ cm^{-2}. The pulsed *L-I* characteristics were measured after the bombardment. In particular, the experimental threshold current densities were correlated with simulated threshold carrier densities to extract the carrier lifetime in each bombarded device.[5]

The experimental *L-I* results shown in Fig. S2 indicate a systematic increase of the lasing threshold with bombardment dose (a sample with the highest dose of $6x10^{13}$ cm^{-2} did not lase at any current). Because the slope efficiency does not vary appreciably with bombardment dose, the simulation neglects any variation of the internal loss with dose. The analysis yields that τ_{car} decreases from 860 ps with no bombardment to 66 ps for a dose of $2x10^{13}$ cm^{-2}. This implies that bombardment of the SA section with a dose of $3x10^{13}$-$1x10^{14}$ cm^{-2} should induce a carrier lifetime in the 10-50 ps range that is intermediate between the pulse length of ≈ 1 ps and the cavity round trip time of ≈ 100 ps. The mode-locked ICLs

discussed in this work were dosed at $5x10^{13}$ cm^{-2}, for which the extrapolated carrier lifetime is ≈ 26 ps.

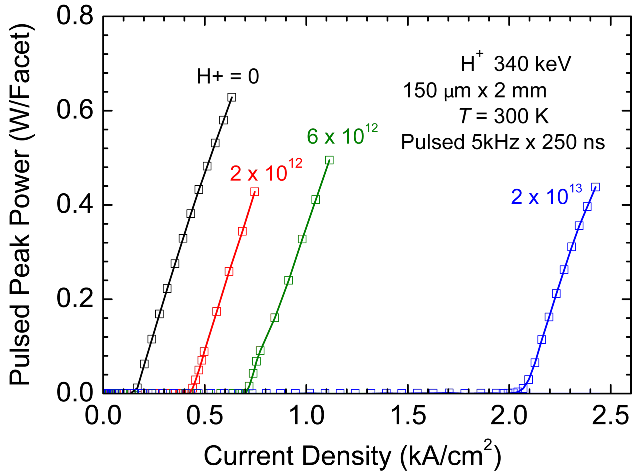

Fig. S2. Pulsed *L-I* characteristics of broad-area ICLs from the same chip that were irradiated with a series of H^+ doses. An additional device bombarded at the highest dose of $6x10^{13}$ cm^{-2} did not lase at all. The carrier lifetime associated with each bombardment dose was extracted by analyzing the threshold current density in conjunction with calculated threshold carrier densities.

2. General characteristics of ICLs with saturable absorber section

Our mode-locked ICL devices feature a split-contact gain/saturable-absorber architecture (Fig.1(b)), in which the top metal contact of the ICL is divided into a longer forward-biased portion and a shorter portion that functions as a saturable absorber (SA) to realize mode locking.

The lasers are biased using a DC current source. and the output power is collected using a high numerical aperture lens (NA=0.85) and then detected on a calibrated thermopile detector. Figure S3(a) illustrates the light-current-voltage characteristics of an ICL cavity with 200 µm contact separation and 100 µm SA contact measured at 15°C when the SA contact is left floating. The threshold current of 85 mA (600 kA/cm^2) is slightly higher than for state-of-the-art ICLs, because the SA section introduces additional optical loss into the cavity.[6] Note also that whereas the *L-I-V* characteristics shown in Fig. S3(a) represent the

average power generated by the mode-locked ICL when a dc bias is applied, most of the output occurs in sub-picosecond pulses with ≈ 100 ps repetition rate, as described above.

Figure S3(b) shows the optical spectra acquired with a Fourier Transform Infrared (FTIR) spectrometer at 15°C under different forward biases and with the SA contact left floating. The spectra show relatively sparse mode structure close to threshold, before expanding into a large number of Fabry-Perot modes at higher bias currents.

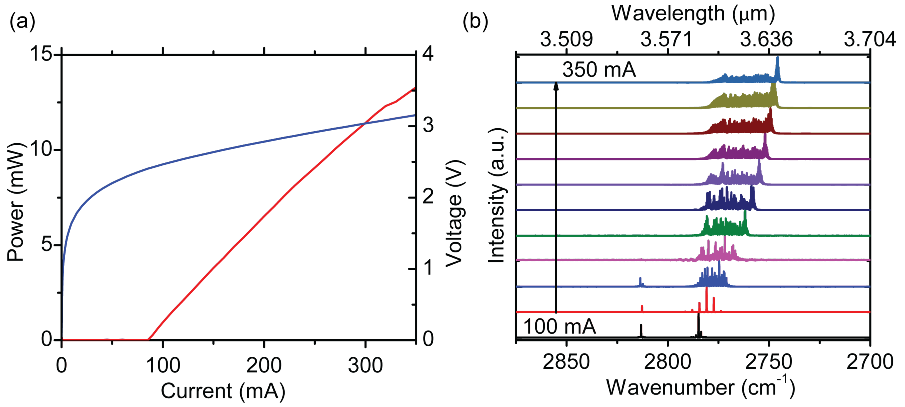

Fig. S3 (a). Collected output power (left axis) and bias voltage of the mode-locked ICL vs. current at $T = 15^{\circ}$C . (b) Lasing spectra of the mode-locked ICL at a series of injection currents (25 mA increments), at 15°C and with the SA junction left floating.

3. Mid-infrared two photon absorption in extended InGaAs photo-detectors

In a linear interband absorption process, an electron-hole pair is generated by the absorption of a photon with energy exceeding the bandgap E_g of the absorbing material. However, when $E_g/2 < h\nu < E_g$, where ν is the photon frequency, a nonlinear process resulting in simultaneous absorption of two photons can also generate an electron-hole pair. The amount of the absorbed radiation scales as with the square of the excitation intensity when the two-photon absorption is relatively weak.[7]

For the mode-locked ICLs emitting at $\lambda \approx 3.6$ μm ($E_g \approx 0.34$ eV), a suitable detector is the extended InGaAs system with bandgap of >1.8 μm. Figure S4(a) shows the setup used to characterize the detector's two-photon absorption properties. The source was a

Fabry-Perot ICL with output collimated by a high-numerical-aperture lens (NA = 0.8). Light emerging from the lens was chopped, and then transmitted through a long-pass optical filter with 2.4 μm cut-on wavelength, to suppress any residual shorter-wavelength emission that could generate linear absorption in the detector. An objective lens focused the collimated light onto a very small spot (a few microns) on the surface of the detector. Finally, the generated photocurrent was analyzed by a lock-in amplifier synchronized to the chopper frequency.

Figure S4(b) plots the lock-in detector's output current as a function of laser input current. The output is seen to increase by > 30 when the input signal increases by 6, which corresponds to a slope of ≈ 2.1 on the log-log scale. This is consistent with a square-law dependence of the detected photocurrent on incident optical power, to within the uncertainty associated with other nonlinear contributions.

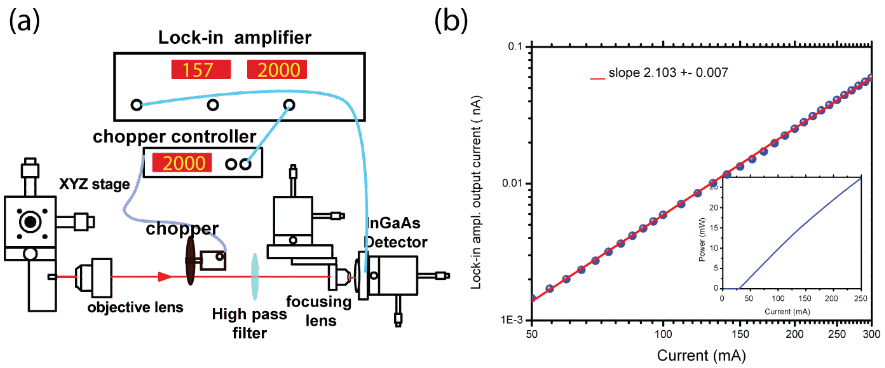

Fig. S4 (a) The setup for measuring two-photon absorption in an extended InGaAs detector. (b) Output current vs. laser input current, showing a slope of 2 in the log-log scale. The inset is the *L-I* characteristic of the ICL used in the testing.

4. Heterodyne beat note experiment

In the heterodyne beat-note experiment arranged in a classic dual-comb setup (see Fig. S5), two free-running mode-locked ICL combs mounted on individual cooling blocks were biased at I = 304 mA (T = 15.4ºC) and I = 287 mA (T = 13.3ºC) using separate low-noise current drivers (Wavelength Electronics QCL500 and ILX LDX-3620). The temperature of each device was kept constant by a multi-channel temperature controller (ILX LDC-3916) that drove the thermoelectric coolers. The laser output beams were collected from the facets

and collimated by AR-coated high-numerical-aperture (NA=0.85) lenses (Thorlabs C037TME-E), and further attenuated using irises to minimize optical feedback. After being overlapped at an AR-coated ZnSe beam splitter, the combined beams were focused by an f/1 off-axis parabolic mirror onto a fast (0.8 GHz 3-dB bandwidth) mid-IR photodetector (HgCdTe, VIGO PVI-3TE-10.6), the AC-coupled output of which was amplified by a 20-dB-gain 1.5 GHz low-noise RF amplifier (HD Communications HD28110) before digitizing. The multi-heterodyne beating waveform was sampled at 20 GS/s, for a duration of 100 μs, by a high-speed 8-bit digital oscilloscope (Lecroy Wavepro 715Zi-A), down-sampled numerically to 2.5 GS/s for vertical resolution enhancement, and post-processed using the procedure described in the next subsection prior to calculating the Fourier Transform. The FTIR data in Fig. S5(b) indicate a broad range of spectral overlap for the outputs from the two mode-locked ICLs.

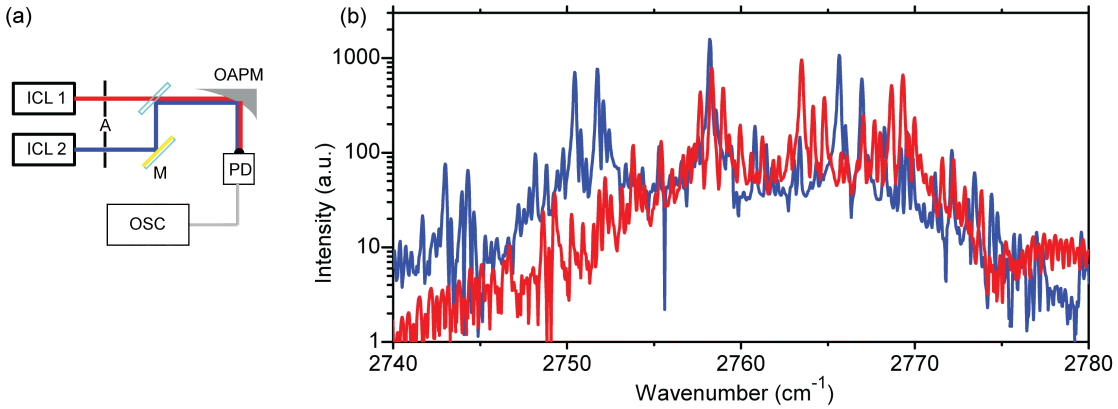

Fig. S5 (a) Experimental setup used in the beat note experiment. ICL1/2 – mode-locked interband cascade laser, A – variable aperture, BS – ZnSe beam splitter, M – mirror, OAPM – off-axis parabolic mirror, PD – fast mid-IR photodetector, LNA – low noise RF amplifier, OSC – high-speed oscilloscope. (b) Log-scale spectra for ICL 1 and ICL 2 when operated under the conditions of the beat-note experiment shown in Fig. 3(a).

5. Coherent averaging of the multi-heterodyne ICL spectrum

Figure 3(c) of the main text shows the coherently averaged electrical spectrum of the time-domain interferogram. It was obtained using a phase-correction technique that was recently demonstrated for multi-heterodyne spectroscopy using noisy but coherent multimode mid-

IR ICL sources[8] (see Reference 8 for complete description) to improve the signal-to-noise ratio of the beat notes. Similar procedures utilizing the Kalman Filter were applied previously in the terahertz, where the authors proved that the greater number of observable RF beat notes is not just a computational artefact, but a real phenomenon with spectroscopic applications[9]. The core of the correction procedure is the assumption that the sources can ultimately be described by two non-stationary parameters, f_{ceo}(t), and f_{rep}(t) ,which define the instantaneous frequency f_n(t) of each optical longitudinal mode:

$$f_n(t) = f_{ceo}(t) + nf_{rep}(t), \tag{S1}$$

Here $f_{ceo}(t)$ denotes the instantaneous carrier-offset envelope (CEO), $f_{rep}(t)$ is the instantaneous repetition rate, and n is an integer. In this model, which is identical to a frequency comb, fluctuations of the CEO frequency shift the mode positions equally, whereas fluctuations of the repetition rate have a mode-number-dependent effect (similar to “breathing”). In multi-heterodyne beating, the down-converted optical signal at a given radio frequency $f_{RF,n}(t)$ follows nearly the same equation, but the parameters are replaced with the difference between the CEO frequencies and the repetition rates:

$$f_{RF,n}(t) = \Delta f_{ceo}(t) + n\Delta f_{rep}(t). \tag{S2}$$

The non-stationary nature of the sources’ optical spectra distributes the amplitude of the down-converted electrical signal over a range of RF frequencies. To counteract this effect, the correction procedure first adaptively resamples the periodic beating signal to equalize the duration of the inferferogram and to compensate for $\Delta f_{rep}(t)$, and then suppresses the common phase noise by shifting the instantaneous phase of the resampled signal to keep $\Delta f_{ceo}(t)$=0.

The assumption of comb-like properties, which may not be true for some incoherent sources, is validated by the algorithm in a natural way. Since it is sufficient to use two RF beat notes to correct for the repetition rate fluctuations, and only one to correct for the CEO

fluctuations, if such a correction yields equal improvement for all modes (both linewidth and amplitude gain), they are mutually coherent. In the beat-note experiment shown in the main text, the signal extracted from two (strongest) beat notes corrects for the available ~90 [Mahmood, something left out here?] and yields approximately 15 dB of gain in the beat note power. It also reduces their apparent linewidths from ~1 MHz to the acquisition time-dependent limit of 10 kHz).

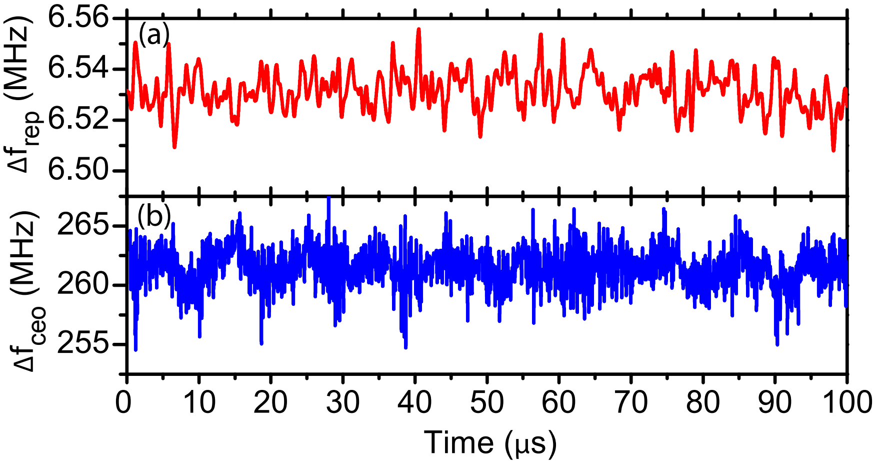

Fig. S6 (a) Retrieved repetition frequency fluctuations and (b) carrier frequency offset fluctuations during measurement.

Figs. S6(a) and (b) show the computationally-retrieved signals, and proves that the jitter in Δf_{ceo}(t) is the main cause of the apparent beat-note broadening to the near-MHz range, as it exceeds that of Δf_{rep}(t) by two orders of magnitude. To illustrate the RF spectrum when no correction is applied, Fig. S7 plots the unprocessed RF spectrum covering 500 MHz. It possesses clearly distinguishable beat notes with SNRs up to 30 dB, which can be improved further by suppressing parasitic optical feedback and electrical noise leaking into the injection current of the lasers. Additionally, the two lasers can be mutually locked by an optical phase locked loop (OPLL), so that one laser tracks the other in the optical domain while preserving a constant position of the beat notes in the RF spectrum with the help of external electronics. Since the lasers in the present experiment were left free running, slight

thermal drifts and electronic noise considerably broadened the beat notes. This drawback can be mitigated in a future system by employing the OPLL to narrow the linewidths.[10]

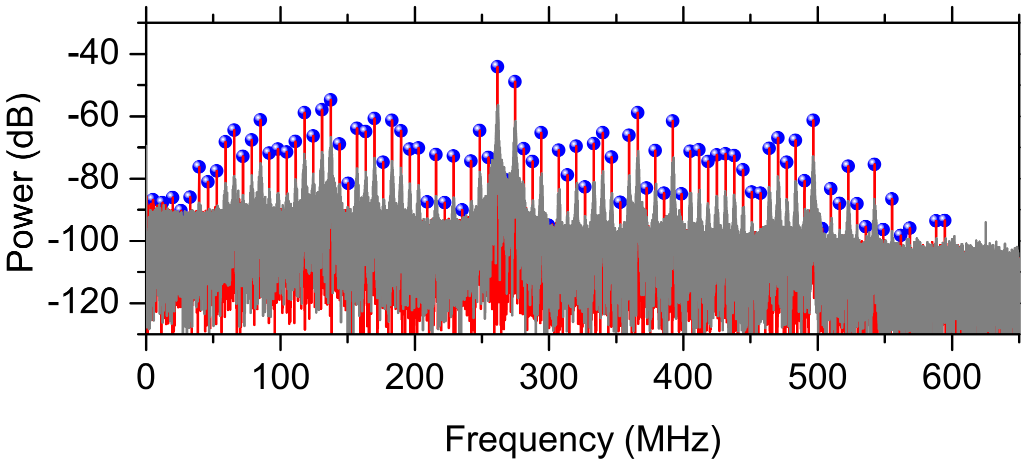

Fig. S7 Comparison between the corrected and non-corrected multi-heterodyne spectra.

6. Time-domain beating signal simulation

Figure 3(d) of the main text shows four periods of the unprocessed time-domain beating signal with significant amplitude modulation. At first glance, this may not resemble a mode-locked laser signal expected to display characteristic bursts of intensity separated by long intervals of nearly zero amplitude. However, a zoom into the central part (Fig. 3(d)) shows good agreement with the simulated waveform $y(t)$, wherein all the M beat notes at discrete frequencies f_i are assumed to have constant root-mean-square amplitudes A_i from the spectrum in Fig. 3(c) of the main text and equal phases ($\Delta\phi$=0),[11] expressed as:

$$y(t) = \sqrt{2}\sum_{i=1}^{M} \mathrm{Re}\,(A_i\, e^{i(2\pi f_i t - \phi_0)}), \tag{S3}$$

where ϕ_0 is the initial phase of the interferogram. The shape of the beating signal in this experiment is determined mainly by the two strongest beat notes around 260-270 MHz, whose power exceeds the average by approximately 25 dB. Equalization of the mode

intensities should yield a time-domain signal more similar to that expected for optical beating between two mode-locked sources.

References

[1] Haus, H. A., *IEEE J. Quantum Electron.,* **11,** 736-746, (1975), “Theory of mode-locking with a slow saturable absorber.”

[2] Van der Ziel, J. P., W. T. Tsang, R. A. Logan, R. M. Mikulyak, and W. M. Augustyniak, *Applied Physics Letters* **39**, 525-527, (1981), “Subpicosecond pulses from passively mode-locked GaAs buried optical guide semiconductor lasers.”

[3] Derickson, D.J., Helkey, R.J., Mar, A., Karin, J.R., Wasserbauer, J.G. and Bowers, J.E., *IEEE Journal of Quantum Electronics*, **28**, 2186-2202, (1992), “Short pulse generation using multisegment mode-locked semiconductor lasers.”

[4] Donetsky, D., Svensson, S. P., Vorobjev, L. E., and Belenky, G., *Appl. Phys. Lett.* **95**, 212104 (2009), “Carrier lifetime measurements in short-period InAs/GaSb strained-layer superlattice structures.”

[5] Bewley, W. W., Lindle, J. R., Kim, C. S., Kim, M., Canedy, C. L., Vurgaftman, I., and Meyer, J. R., *Appl. Phys. Lett.* **93**, 041118 (2008), “Lifetimes and Auger Coefficients in Type-II “W” Interband Cascade Lasers.”

[6] Forouhar, S., Borgentun, C., Frez, C., Briggs, R.M., Bagheri, M., Canedy, C.L., Kim, C.S., Kim, M., Bewley, W.W., Merritt, C.D., Abell, J., Vurgaftman, I., and Meyer, J. R. *Applied Physics Letters*, **105**(5), (2014), “Reliable mid-infrared laterally-coupled distributed-feedback interband cascade lasers.”

[7] Hayat, A., Nevet, A., Ginzburg, P. and Orenstein, M., *Semicond. Sci. Technol.* **26**, 083001 (2011), "Applications of two-photon processes in semiconductor photonic devices."

[8] Sterczewski, L. A., Westberg J., Patrick, L., Kim, C. S., Kim, M., Canedy, C. L., Bewley, W. W., Merritt, C. D., Vurgaftman, I., Meyer, J. R., and Wysocki, G., *Opt. Eng.* **57**(1), (2017), “Multiheterodyne spectroscopy using interband cascade lasers.”

[9] Burghoff, D., Yang, Y. and Hu, Q., *Sci. Adv.* **2**, 11, (2016), “Computational multiheterodyne spectroscopy.”

[10] Westberg, J., Sterczewski, L. A., and Wysocki, G., *Applied Physics Letters*, **110**, (2017) “Mid-infrared multiheterodyne spectroscopy with phase-locked quantum cascade lasers.”

[11] Barbieri, S., Ravaro, M., Gellie, P., Santarelli, G., Manquest, C., Sirtori, C., Khanna, S. P., Linfield, E. H., and Davies, A. G., *Nat. Photon.* **5,** 5 (2011), "Coherent Sampling of Active Mode-Locked Terahertz Quantum Cascade Lasers and Frequency Synthesis."
